# Choriorétinite extensive bilatérale révélant une infection par le virus de l'immunodéficience humaine (VIH)

**DOI:** 10.11604/pamj.2014.19.65.4775

**Published:** 2014-09-24

**Authors:** Oubaida El Yamouni, Nazih Tzili, Abdallah El Hassan, Nadia Slassi, Mahfoud El Khaoua, Zakaria Jebbar, Amina Berraho

**Affiliations:** 1 Service d'Ophtalmologie B, Hôpital des Spécialités, CHU Rabat, Maroc

**Keywords:** Choriorétinite, virus de l′immunodéficience humaine(VIH), Sulfadiazine, pyriméthamine, Ganciclovir, chorioretinitis, HIV, Sulfadiazine, pyrimethamine, Ganciclovir

## Abstract

Au cours de l'infection par le virus de l'immunodéficience humaine(VIH), Les atteintes oculaires sont polymorphes, pouvant compromettre le pronostic fonctionnel. Nous rapportons l'observation d'un patient présentant une choriorétinite infectieuse sévère révélant une infection par le VIH. Patient âgé de 35 ans avec antécédent de tuberculose pulmonaire en 2007, consulte pour BAV bilatérale progressive depuis 6 mois. Une acuité visuelle à compte les doigts au niveau de l'oeil droit et à mouvement des doigts au niveau de l'oeil gauche, avec présence de foyers choriorétiniens diffus visualisés au fond d'oeil et à l'angiographie. Les sérologies VIH, toxoplasmose et CMV sont positives. Le patient a été mis sous traitement anti-toxoplasmose (Sulfadiazine et pyriméthamine) et anti-CMV (Ganciclovir per os). L’évolution sous traitement a été marquée par une régression de la hyalite avec la persistance des foyers choriorétiniens évolutifs et une acuité visuelle réduite à perception lumineuse.

## Introduction

L'infection à VIH touche actuellement 39,5 millions de personnes à travers le monde, dont 90% vivent dans les paysen voie de développement. 50% à 100% des patients infectés par le VIH présentent une atteinte oculaire au cours de la maladie [1, 2]. Nous rapportons l'observation d'un patient présentant une choriorétinite infectieuse sévère révélant une infection par le VIH.

## Patient et observation

**Figure 1:**
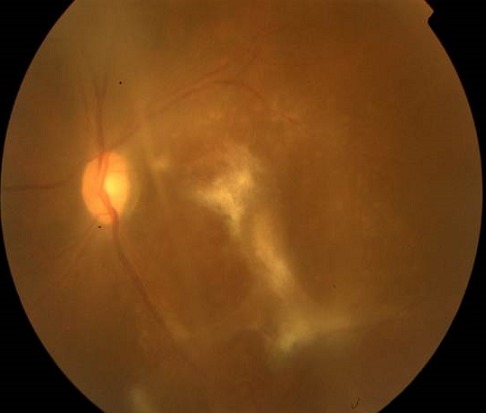
Photo du fond d’œil gauche: Large foyer blanchâtre dans l'aire maculaire s’étendant vers 6h

**Figure 2:**
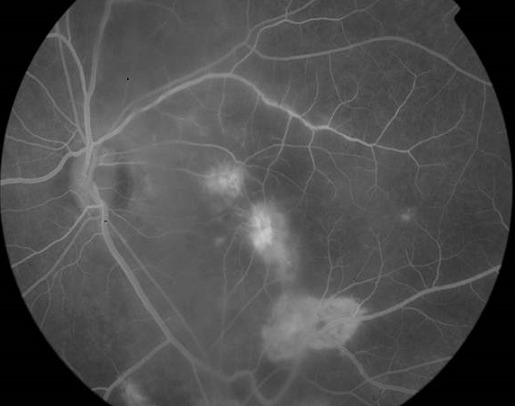
Cliché après injection de la fluorescéine: foyer blanchâtre maculaire et paramaculaire de fluorescence hétérogène dont l'un centré par un vaisseau

**Figure 3:**
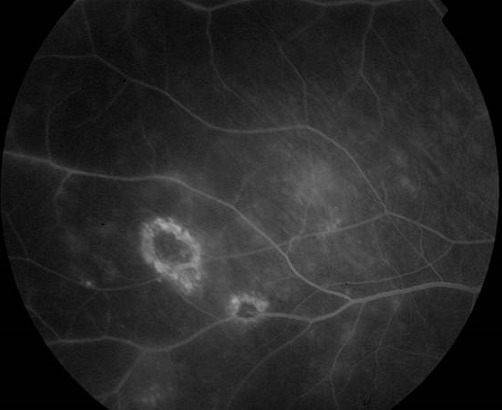
2 foyers cicatriciels avec prise de fluorescéine périphérique

**Figure 4:**
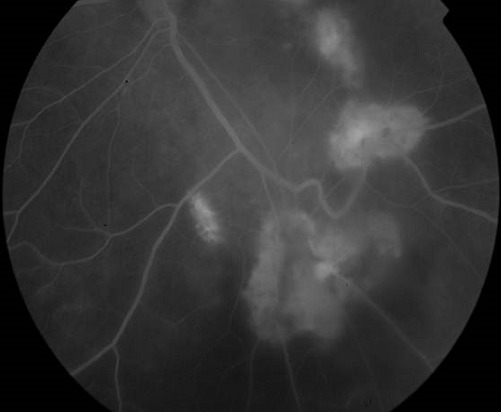
Foyers choriorétiniens d'allure évolutifs avec prise hétérogène de fluorescéine

Il s'agit d'un patient âgé de 35 ans, avec un antécédent de tuberculose pulmonaire en 2007 qui consulte pour une baisse de l'acuité visuelle bilatérale progressive depuis 6 mois avec aggravation il y a 1 mois. L'examen clinique au niveau de l'oeil droit trouve une acuité visuelle à compte les doigts à 2 mètres inaméliorable, une hyalite à 2 croix, et au fond d'oeil 3 foyers blanchâtres légèrement pigmentés à 6h, 9h et 11h, 2 foyers blanchâtres à 1h et 2h avec de multiples trous entre ces 2 foyers. Au niveau de l'oeil gauche on trouve une acuité visuelle à mouvement des doigts, hyalite à 2 croix, et au fond d'oeil 1 foyer au pôle postérieur allant de la papille vers 6h prenant la macula, puis 2 foyers en placards blanchâtres de gliose à 10h et 11h associées à une vascularite diffuse et une papillite (Figure 1). L'angiographie à la fluorescéine trouve à l'oeil gauche un large foyer blanchâtre dans l'aire maculaire s’étendant vers 6h, et des foyers choriorétiniens d'allure évolutifs avec prise hétérogène de fluorescéine (Figure 2, Figure 3, Figure 4). Les sérologies VIH, toxoplasmose et CMV sont positives. Le patient a été mis sous traitement anti-toxoplasmose (Sulfadiazine et pyriméthamine) et anti-CMV (Ganciclovir per os). L’évolution sous traitement a été marquée par une régression de la hyalite avec la persistance des foyers choriorétiniens évolutifs et une acuité visuelle réduite à perception lumineuse.

## Discussion

Les atteintes au cours du SIDA sont polymorphes. Les rétinites à CMV sont les infections les plus fréquentes et de loin la cause la plus responsable de cécité chez les malades atteints de VIH [3]. La rétinite à CMV réalise une nécrose extensive de la rétine [4] et se présente sous forme d′une plage nécrotique blanchâtre centrée par un vaisseau. Dans 40% des cas, elle ne s'accompagne à son début d'aucun signe fonctionnel, ce qui justifie le suivi ophtalmologique systématique des patients ayant le sida [5, 6]. La rétinite toxoplasmique est la deuxième cause de rétinite chez le patient séropositif au VIH. Elle se présente comme un foyer blanchâtre du pôle postérieur avec inflammation du vitré. À la différence du sujet sain où le toxoplasme se localise dans le tissu rétinien, il a plutôt une localisation choroïdienne en cas de sida [7]. Toutes ces lésions ont été retrouvées chez notre patient, les unes évolutives et les autres au stade de séquelles.

Le traitement curatif de la choriorétinite à CMV repose sur le Ganciclovir et le foscarnet; celui de la choriorétinite toxoplasmique fait appel à la pyriméthamine et la sulfadiazine. Le pronostic fonctionnel est réservé dans la plupart des séries décrites; notre patient garde une acuité visuelle à une perception de lumière malgré un traitement d'un mois. Depuis 1996, grâce à la multi thérapie antirétrovirale, les infections opportunistes associées au SIDA sont en nette régression [8]; Cependant, il existe toujours des formes sévères de choriorétinites comme c'est le cas de notre malade qui présente comme signe de sévérité une co-infection; une atteinte maculaire et un retard de prise en charge.

## Conclusion

Les rétinites à cytomégalovirus et toxoplasmique représentent une complication oculaire majeure au cours de l’évolution du SIDA. Le pronostic visuel reste toutefois réservé malgré l'instauration d'un traitement anti-infectieux adéquat.
